# Indicators for Healthy Ageing — A Debate

**DOI:** 10.3390/ijerph10126630

**Published:** 2013-11-29

**Authors:** Judith Fuchs, Christa Scheidt-Nave, Timo Hinrichs, Andreas Mergenthaler, Janine Stein, Steffi G. Riedel-Heller, Eva Grill

**Affiliations:** 1 Department of Epidemiology and Health Monitoring, Robert Koch Institute, General-Pape-Str. 62-66, D-12101 Berlin, Germany; E-Mail: Scheidt-NaveC@rki.de; 2 Swiss Paraplegic Research, Guido A. Zaech Strasse 4, CH-6207 Nottwil, Switzerland; E-Mail: timo.hinrichs@paraplegie.ch; 3 Department of Sports Medicine and Sports Nutrition, University of Bochum, D-44801 Bochum; Germany; 4 Bundesinstitut für Bevoelkerungsforschung (BiB), Federal Institute for Population Research, Friedrich-Ebert-Allee 4, D-65185 Wiesbaden, Germany; E-Mail: andreas.mergenthaler@bib.bund.de; 5 Institute of Social Medicine, Occupational Health and Public Health (ISAP), Medical Faculty, University of Leipzig, Philipp-Rosenthal-Straße 55, D-04103 Leipzig, Germany; E-Mails: Janine.Stein@medizin.uni-leipzig.de (J.S.); Steffi.Riedel-Heller@medizin.uni-leipzig.de (S.G.R.-H.); 6 Institute for Medical Informatics, Biometry and Epidemiology (IBE), Ludwig-Maximilians-Universitaet Munich, Marchioninistr. 15, D-81377 Munich, Germany; E-Mail: Eva.Grill@med.uni-muenchen.de

**Keywords:** old age, health, Germany, methodology

## Abstract

Definitions of healthy ageing include survival to a specific age, being free of chronic diseases, autonomy in activities of daily living, wellbeing, good quality of life, high social participation, only mild cognitive or functional impairment, and little or no disability. The working group Epidemiology of Ageing of the German Association of Epidemiology organized a workshop in 2012 with the aim to present different indicators used in German studies and to discuss their impact on health for an ageing middle-European population. Workshop presentations focused on prevalence of chronic diseases and multimorbidity, development of healthy life expectancy at the transition to oldest-age, physical activity, assessment of cognitive capability, and functioning and disability in old age. The communication describes the results regarding specific indicators for Germany, and hereby contributes to the further development of a set of indicators for the assessment of healthy ageing.

## 1. Introduction

Demographic prognoses for Europe project that the proportion of older people within the general population will steadily rise from an estimated percentage of population aged 65 years and over of 16.0% in 2010 to a projected value or 29.3% in 2060 [1,2] This will as well lead to an increase in the number of people with limitations with considerable effects on social security systems, public health, and society. For many years, researchers have emphasised that getting older is not necessarily a precondition of morbidity. Rowe and Kahn [3] introduced the concept of successful ageing, separating the effects of disease from the process of ageing. Since then, a rapidly growing body of literature has been investigating the components of how people age successfully or healthily, confirming the multidimensional character of the ageing process [4]. The WHO defines active ageing as “the process of optimising opportunities for health, participation, and security in order to enhance quality of life as people age” [5]. To put this into perspective, successful, active, or healthy ageing includes the notion of successful adaptation to changes that cannot be prevented [6].

Why is a valid definition of healthy ageing so important?

**Figure 1:**
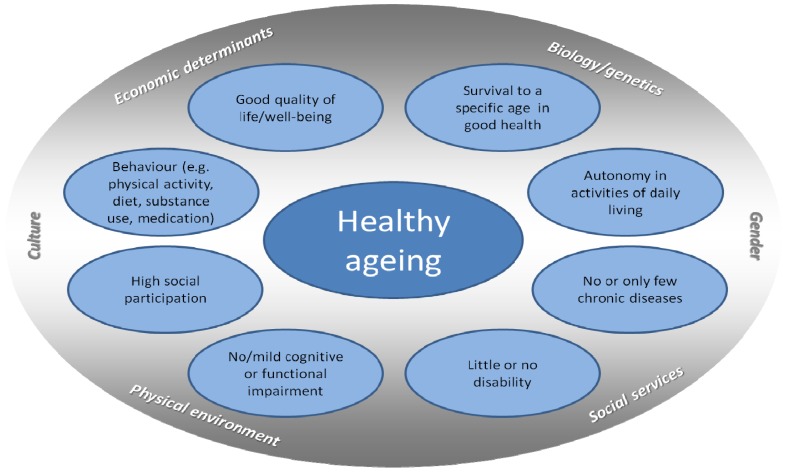
Components of healthy ageing.

The impact and importance of predictors, risk factors, and preventive interventions can only be evaluated if the outcome has been defined beforehand. Peel, Bartlett and McClure [6] showed in their review that, due to the lack of a univocal definition, there is not even a clear idea about the prevalence and incidence of healthy ageing. To give an example, domains typically included in definitions of healthy ageing are survival to a specific age, being free of chronic diseases, autonomy in activities of daily living, wellbeing, good quality of life, high social participation, only mild cognitive or functional impairment, and little or no disability. Figure 1 shows an overview on the different components. 

Today, the term “healthy ageing” is widely used and comprises a broad variety of indicators and predictors. Fernandez-Ballesteros*, et al.*, [7] consider the terms active, healthy, successful or productive aging as strongly related and as multidimensional concepts, referring to a positive way of ageing, but underline, that a commonly accepted definition is still missing.

While all of these positive outcomes are objectives of high importance to both the individual and the society, measuring healthy ageing as an outcome becomes difficult and depends on the definition and weighting of its components [8]. This can be illustrated by looking at each of the components:

To start with the most obvious, the study of morbidity in old age is one of the most dominant topics. However, there is no consensus on whether increased life expectancy also increases the number of years lived with disease or what this might signify [9,10,11,12]. Characteristic of older age is the presence of multiple conditions, known as multimorbidity (MM) [13]. Prevalence of MM is strongly related to the type and number of conditions entering this summary measure [14].

Secondly, regarding disability, Robine and colleagues stated in 2004 that a general theory of population ageing and disability transition is still missing [12]. They proposed moving forward by setting up universal measures for functioning and disability. In the meantime widely used sets of instruments, as the NIH Toolbox [15,16] are available, but nonetheless, there is still a need for standardisation 

Thirdly, the concept of frailty, a state of increased vulnerability, [17] has gained much popularity because it includes multiple measures of biological ageing into a summarised measure. Also, components of frailty such as grip strength or walking speed have been shown to be highly predictive of mortality. Still, this concept is not univocally agreed upon; a recent Delphi exercise stated that there is not one valid definition of frailty but many [18]. Frailty could be considered as a counterpart of healthy ageing.

Finally, quality of life and social participation as components of healthy ageing are highly subjective issues and largely depend on personal and environmental factors. Actual objective participation has to be differentiated from subjective or desired participation. 

Returning to the question of what constitutes healthy ageing, all the components described above are preconditions for independent living but hardly meet the requirements for a valid definition. We are thus still at the point where indicators of healthy ageing have to be discussed and evaluated. Much of the recent activity in gerontology research focuses on diseases and deficits and not on potentials and resources for healthy ageing. These aspects of healthy ageing could be addressed by targeted interventions. 

International programs like the European Innovation Partnership on Active and Healthy Ageing [19] were established in order to collect information on factors for healthy ageing. The European Union created the campaign European Year for Active Ageing and Solidarity between Generations 2012 (EY 2012) to move towards the goal of the Age-Friendly European Union by 2020. This implies important individual and societal goals such as minimising impairments and limitations, living actively, and participating in society. Several European and national collaborative research programs like the Survey of Health, Ageing, and Retirement in Europe (SHARE) [20], the British program Healthy Ageing across the Life Course (HALCYON) [21], or the German research initiative on Health in Old Age [22] contribute data to help describe challenges in European countries.

With this in mind, the working group Epidemiology of Ageing of the German Association of Epidemiology organised a workshop in the autumn of 2012 that collected evidence on indicators of healthy ageing. We focused on five specific domains:

- Prevalence of chronic diseases and multimorbidity;
- Development of healthy life expectancy at the transition to oldest-age;
- Physical activity in old age;
- Assessment of cognitive capability in old age;
- Functioning and disability in old age.

The objectives of this paper are to put forth the results of the presentations, to describe results regarding specific indicators for Germany, and to create a basis for the further development of indicators of healthy ageing that are relevant for all areas of epidemiological research. Our specific aim is to discuss feasibility and practicality of existing indicators for different epidemiological scenarios—namely cohort studies, health reporting, and health services research.

## 2. Results

### 2.1. Prevalence of Chronic Diseases and Multimorbidity

Freedom from major illness [3] is one of many definitions of healthy ageing. The absence of chronic conditions can be determined by estimating prevalences of single conditions or co- and multimorbidity (MM). MM is very common in old age [13], and the challenge for healthcare systems is to consider individual potentials and limitations due to the present health situation. Furthermore, chronic diseases and MM may differ between birth cohorts, social status, or regions, and time trends. These factors need specific consideration when carrying out analyses as confounders and mediators may differ.

Analyses from a German health survey showed how prevalence of MM differs by age [23], but conclusions were limited due to small numbers of participants in older age-groups. For the workshop we used pooled data from two nationally representative health surveys funded by the German Federal Ministry of Health in 2009 (GEDA 2009) and 2010 (GEDA 2010) to describe the prevalence and patterns of morbidity in older and old people in Germany. Participants (43,312) aged 18 to 100 years completed a computer-assisted telephone interview (CATI). Both surveys covered a range of health topics, and health-related and sociodemographic variables. We analysed data from 8,179 people (57.8% women) aged 65 to 100 years old and estimated age- and sex-specific prevalences for single health conditions, disease categories, and MM. 

We included 16 medical diagnoses (lifetime prevalences for stroke, myocardial infarction (MI) or other coronary heart disease, any type of malignant disease, 12-month prevalence for hypertension, hyperlipidemia, diabetes mellitus, chronic heart failure, asthma, chronic bronchitis, chronic renal disease, chronic liver disease, osteoarthritis, rheumatoid arthritis, osteoporosis, depression, and current medication use for hypertension or hyperlipidemia), three health problems (self-reported chronic back pain and self-reported severe hearing or vision impairment), and obesity (BMI ≥ 30 kg/m^2^ based on self-reported height and weight). Health conditions were grouped into 9 disease categories defined by organ systems or disease entities. MM was defined as having two or more out of 20 conditions or health problems.

The most prevalent single health conditions for participants aged 65 to 100 years were hypertension (55.1%), hyperlipidemia (40.4%), osteoarthritis (38.8%), chronic back pain (30.4%), and obesity (20.3%). Health conditions with gender-specific high prevalence were coronary heart disease in men (25.1%) and osteoporosis in women (20.2%). One should consider that the related burden of disease of single conditions highly depends on treatment and present complaints. The two most prevalent disease categories were cardiometabolic and musculoskeletal conditions. 

The majority of participants reported MM; it was present in 78.1% of women and 70.1% of men. Those with low social status showed significantly higher prevalence of MM. Focusing on healthcare services utilisation, we found that people with MM use significantly more ambulatory and inpatient care than persons without MM. The mean number of doctor visits was 8.3 *vs.* 3.9; the mean number of hospital nights was 3.7 *vs.* 1.3. 

When addressing MM, it is important to emphasise that the methodology for systematic assessment and analysis needs to be nationally and internationally agreed upon [14]. Cross-sectional and longitudinal analyses of MM should consider the context of participation, functional limitations, disability, frailty, quality of life, and autonomy.

### 2.2. The Development of Healthy Life Expectancy at the Transition to Oldest-Age

Life expectancy at older ages is steadily increasing in Germany [24]. Whether this trend is accompanied by an increase of time spent in good health is a controversial issue [10,25]. With this background, we examined if an increase in health expectancy as predicted by the “compression of morbidity” hypothesis [26] can be observed since the mid-1990s among the German population beyond retirement age. Moreover, we examined to what extent different trends in health expectancy among the elderly can be explained by social, economic, and demographic determinants.

The analyses were based on two birth cohorts of the German Ageing Survey (DEAS) born between 1911 and 1926 and between 1917 and 1932 [27]. Respondents that were 70 years and older at baseline (1996 and 2002, respectively) were selected. Data on life expectancy from the period life tables of the German Federal Statistical Office were linked to the survey data of the DEAS. Sullivan’s method [28] was employed to calculate health expectancy. The data from the period life tables were weighted by the age- and sex-specific prevalence of “good” subjective health in each of the two birth cohorts of the DEAS. A “good” subjective health was defined as respondents reporting a “very good”, “good”, or “fair” level of health. To adjust for confounding, the sex of the respondents, their place of residence (Eastern *vs.* Western Germany), and their marital status was considered. Additionally, the analysis adjusted for socioeconomic characteristics like highest educational degree, the equivalent income of the respondents’ household, residential property and car ownership, and assets. Social capital was integrated into the analysis by the degree of interaction with the neighbours, the relatedness to the community, and the respondents’ fear of crime. To examine the impact of those confounders on the association between birth cohort and health expectancy, multiple random coefficient models (growth curve models) [29] were calculated.

Descriptive analysis reveals that males from the birth cohort from 1917 to 1932 could expect 6 years and 8 months in “good” health compared to 6 years and 4 months among males in the birth cohort from 1911 to 1926. With regard to women, the results point towards an expansion of morbidity since the older cohort (born between 1911 and 1926) could expect 7 years in “good” health, whereas women in the younger cohort could expect 2 months less. The full model confirms these findings. The fixed effect parameter for health expectancy of the younger birth cohort (1917 to 1932) was slightly higher compared to the cohort born between 1911 and 1926. The association remained statistically significant after controlling for demographic characteristics, socioeconomic resources, and social capital (estimate for the cohort born between 1911 to 1926 compared to the younger cohort born between 1917 to 1932: −0.17, *p* < 0.1%). However, the effect was strongly moderated by sex and residential region (estimates for the interactions of sex and residential region by birth cohort: −0.20, *p* < 0.1% and 0.15, *p* < 0.1%, respectively). For men living in Western and Eastern Germany, a compression of morbidity could be observed. This trend was more pronounced for Eastern Germany, where men from the younger cohort could expect to live over 7 months longer in “good” subjective health compared to men from the older cohort. For Western German men, this difference only accounted for 3 months. In contrast, there was no significant difference between cohorts for women in Eastern Germany. Moreover, the health expectancy for the younger cohort of Western German women was about 6 months lower compared to women from the older cohort. This finding suggests an expansion of subjective morbidity among women in the western federal states of Germany.

To calculate health expectancies, the prevalence-rate life-table model (or Sullivan’s method) was employed. However, there is on-going debate as to whether this method provides unbiased estimates of time trends in health expectancies [25,30]. Since there were only two waves of the DEAS available for each birth cohort, the number of observations was comparatively low. Thus, no complex methods like multistate life tables were used to estimate health expectancies.

The cases included in the study cohorts were mutually exclusive. This lowered the number of cases and observations (392 observations in the birth cohort 1917 to 1932 and 472 observations in the birth cohort 1911 to 1926) in the study cohorts and constrained the power of the statistical analysis. However, compared to the cross-sectional data of the DEAS, the information obtained by analysing a longitudinal sample with random coefficient models was still higher, as indicated by a statistically significant intraclass correlation of 72% (data not shown). 

In contrast to measures of Disability-Free Life Expectancy (DFLE) or Disability-Adjusted Life Expectancy (DALE), which are based on indicators of disease and functional impairment [31], the current study employed the subjective health of the respondents to estimate the healthy life expectancy. DFLE or DALE could not be calculated using the DEAS data because indicators of functional health were not measured in a similar way in each wave. Subjective health provides an indicator of a subject’s health-related quality of life and autonomy in older age groups. Furthermore, one of our indicators of healthy life expectancy based on subjective health is comparable to empirical evidence from Austria [32].

The study reveals that the trends in health expectancy among elderly people in Germany are heterogeneous with regard to sex and region. These results further support the conclusion that the dynamics of health expectancies are stratified across multiple sociodemographic, spatial, and socioeconomic factors [25]. Given the limitations of the data employed, further research is needed to disentangle the underlying mechanisms that account for the observed differences in health expectancy. 

### 2.3. Physical Activity in Old Age

Epidemiological research suggests that regular physical activity (PA) is closely related to the health of older adults. This includes an inverse association with various chronic diseases [33] as well as with functional deficits such as limitations in activities of daily living and mobility [34,35]. In addition, PA seems to be positively correlated with psychological constructs such as self-efficacy, quality of life, and wellbeing [33]. PA generally decreases with age, and most older adults are insufficiently active compared to current PA recommendations [36]. In light of the public health burdens associated with sedentary behaviour in old age [37], it is important to understand why some older adults are less active than others and to derive starting points and recommendations for targeted public health strategies aiming to increase PA levels of older populations.

To contribute to the latter aim, a series of exploratory cross-sectional studies were set up in a cohort of community-dwelling older adults (getABI) within the research consortium PRISCUS [38]. The main objectives were: (**1**) to analyse PA patterns and to evaluate factors associated with PA in different domains (*i.e.*, sporting *vs.* domestic activities) stratified by gender [39], (**2**) to analyse barriers to PA stratified by gender and by age group [40], and (**3**) to evaluate the rate of older patients receiving advice on PA from their general practitioner (GP) [41].

The “German Epidemiological Trial on Ankle Brachial Index” (getABI) is a prospective cohort study. Its design and methods have been described elsewhere in detail [42]. In order to collect comprehensive data on PA in the 7-year follow-up of the getABI cohort, a short instrument to measure PA by telephone interview was needed. For this purpose, the 10-item PRISCUS-Physical Activity Questionnaire (PAQ) was developed. Prior to its application in the getABI cohort, precursor studies showed an acceptable reliability and validity of the PRISCUS-PAQ [43,44]. At the time of the 7-year follow-up telephone interview, all participants of the getABI cohort were at least 72 years old. Men spent more time with sporting activities while women performed more domestic activities (including heavy housework and gardening). The need of a walking aid lowered the odds of being active in both activity domains. Being interviewed in spring or summer increased the chance of performing domestic activities [39]. The most frequently reported barrier to PA was poor health (58% of participants), especially in the age group 80+ (71%) [40]. About two thirds of participants had not been advised by their GP to get more physically active within the past 12 months [41].

By applying the newly developed PRISCUS-PAQ to the getABI cohort, a database for PA of older adults in Germany was created. The exploratory studies showed gender-related differences and seasonal variations in PA behaviour [39]. Mobility limitations and health problems were identified as major barriers to PA, especially among the “oldest old” [40]. While GPs are supposed to play an important role in promoting PA to their older patients, the present analyses revealed a relatively low rate of participants actually receiving advice on PA from their GP [41]. The delineated subgroup differences in PA patterns and barriers not only have implications for future PA promotion and intervention strategies (e.g., target group-specificity/individualisation) but also for future epidemiologic research (e.g., stratification by gender/age group where applicable; possible bias by seasonality of PA behaviour). 

### 2.4. The Assessment of Cognitive Capability in Old Age

Results from the German Study on Ageing, Cognition, and Dementia in Primary Care Patients (AgeCoDe Study) were reported to give an overview of the assessment of cognitive changes in the elderly via neuropsychological instruments and the usage of change norms. What are change norms and why are they so urgently needed? Generally, healthy ageing and normal ageing processes are accompanied by non-pathological cognitive decline. In contrast, substantial cognitive deterioration over time represents a diagnostic criterion for dementia diseases [45]. Improvements in cognitive capabilities could in turn reflect the beneficial effects of affective therapy or treatment. In clinical routine and research settings, screening tests and neuropsychological instruments are a crucial and indispensable part of the objective assessment of cognitive performance and cognitive changes [46]. Against this background, the differentiation between normal age-associated changes and pathological cognitive decline or significant cognitive improvement often represents a difficult task for geriatricians, neurologists, or neuropsychologists. The simple comparison between pre- and post-test scores is too limited and does not take into account probable measurement error, practice effects, or other biasing factors [47]. Reliable Change Indices (RCIs) represent a group of statistical methods for calculating longitudinal normative data (*i.e.*, change norms) in order to interpret changes in cognitive functioning over time as measured by psychometric tests [48]. Principally, RCIs allow the intra-individual comparison between a person’s achieved cognitive test performance and the test performance that would be expected to be achieved in the cognitively healthy elderly population. There is a rising need for RCIs for many screening tests, the calculation of change norms, and neuropsychological instruments to assess and track cognitive capability in the elderly population [49]. 

Research shows that cognitive test performance is influenced by certain factors including sociodemographic variables (e.g., age, education, and gender) and other lifestyle variables or health conditions (e.g., nutrition, MM, and physical activity) [50]. Moreover, certain risk and protective factors were identified as having an impact on cognitive functioning in old age and the development of dementia. Higher educational level and mental activity across the life span, for example, are associated with a lower risk of developing dementia. This protective link is mostly attributed to an increased cognitive reserve capacity that compensates deficits occurring in dementia [51,52]. Thus, the calculation of change norms and RCIs should take these factors into account [49]. 

The AgeCoDe study offered an excellent opportunity and data basis to calculate change norms in terms of RCIs for neuropsychological instruments for the assessment of cognitive capability in old age. The AgeCoDe study is a multicentre, prospective longitudinal study on the early detection of mild cognitive impairment and dementia in primary care. The study was founded by the German Research Network on Dementia and started in 2003. Study participants were recruited via general practitioners (GPs). Inclusion criteria were being 75 years of age or older, the absence of dementia, and at least one contact with the GP within the last 12 months. Currently, the study is carrying out the 6th follow-up assessment in six study centres in Germany (Bonn, Düsseldorf, Hamburg, Leipzig, Mannheim, and Munich). Consultations with participants included comprehensive standardised face-to-face clinical interviews and neuropsychological assessments in the subjects’ home environment at regular intervals of 1.5 years. The main instruments used to assess cognitive functions were the Structured Interview for the Diagnosis of Dementia of the Alzheimer Type, Multi-infarct Dementia and Dementia of other Etiology according to DSM-III-R, DSM-IV and ICD-10 (SIDAM), the Mini-Mental State Examination (MMSE), and the CERAD-NP battery. Within this study, RCI scores were calculated for all three instruments [53,54,55]. The RCI scores were adjusted for the sociodemographic variables of age, gender, and education as these variables showed a significant impact on cognitive test performance. Altogether, the age-, education- and gender-specific RCI scores represent a valuable contribution to the reliable assessment of cognitive changes in older adults. In the future, there should be continued efforts to develop and to apply RCI scores for established psychometric instruments in order to optimise the evaluation of cognitive capability in the elderly population.

### 2.5. Functioning and Disability in Old Age

Disability denotes all impairments, activity limitations, and participation restrictions [56] that are often operationalized by restrictions in activities of daily living and mobility. Conversely, functioning describes the positive aspects of these concepts. A person’s health status, as well as personal and contextual characteristics such as family support, influences his or her functioning. In the context of ageing research, disability is largely defined as a limitation in basic activities of daily living *i.e.*, basic care needs such as washing, dressing, or eliminating, or as a limitation in instrumental activities of daily living such as shopping, lifting and carrying, gardening, or driving.

The KORA-Age survey, funded within the German research initiative on “Health in Old Age” [57], was initiated to answer questions on determinants of successful ageing and MM in the aged in Germany. Data of 4,117 participants aged 65 and above were used to analyse the prevalence and determinants of disability [58]. Disability was assessed by telephone interview with the Health Assessment Questionnaire Disability Index (HAQ-DI) [59]. Minimal disability, defined as any restriction in one or more of the domains of the HAQ-DI, was highly prevalent in all age groups. Prevalence of disability increased with age, with a very steep and abrupt increase in participants over 80. Female sex, lower per capita income, physical inactivity, and malnutrition were factors significantly associated with disability when adjusting for age and morbidity. Stroke and neurological diseases were strongly associated with disability, but joint and eye diseases contributed most to the burden of disability in this group.

However, we have to acknowledge that a single disease may have more than one consequence and that individuals may present with symptoms, impairment of function, or restriction of activity unexplained by the underlying pathology. In addition, the WHO’s biopsychosocial model underlines the importance of contextual factors, both physical and social environment and personality traits, and factors such as self-efficacy, resilience, and biography. It seems obvious that healthy ageing is easier in a welcoming and empowering environment, and that it may equally depend on personal traits, attitudes, and resources. 

## 3. Conclusions

Various indicators for healthy ageing are used in German studies on ageing. This inaugural meeting of the Epidemiology of Ageing Group opened the dialogue for German epidemiologists about parity between measurement tools in population studies The studies presented during the workshop showed, that a variety of indicators are used in different population-based studies in Germany. Many are feasible and reliable for the description of different components of healthy ageing. Further studies on healthy ageing are encouraged to including one or several of the presented indicators in order to obtain comparable data. Furthermore, results emphasise the importance of a multifactorial approach, including a comprehensive assessment of resources, diseases and complaints, cognitive and functional capacities, and limitations and disability while also giving consideration to environmental, sociodemographic, and socioeconomic factors and biological/genetic determinants. Analysis of healthy ageing must take this complexity into account. The discussion came up with a broad agreement that the debate should be continued in order to identify an agreed-upon basic set of instruments for research regarding people of older age in Germany compatible with international studies. We recognise that not all the measurement tools included in this debate may be appropriate for all studies; however, a joint effort should be made to harmonize measures as much as possible.

As a key outcome of the meeting of the Epidemiology of Ageing group we underline that further work is required to achieve a core set of indicators in epidemiological studies in Germany. All members agreed to continue with annual workshops and closer networks to achieve this aim. By discussing the on-going studies and numerous measurement tools used we will continue to work together to ensure that life course epidemiology in Germany aligns with international efforts. 
